# Raphe-Hippocampal Serotonin Neurotransmission In The Sex Related Differences of Adaptation to Stress: Focus on Serotonin-1A Receptor

**DOI:** 10.2174/157015911796558019

**Published:** 2011-09

**Authors:** Darakhshan Jabeen Haleem

**Affiliations:** Department of Biochemistry, Neurochemistry and Biochemical Neuropharmacology Research Unit, University of Karachi, Karachi 75270, Pakistan

**Keywords:** Raphe, hippocampus, sex related differences, stress 5-HT-1A receptors, serotonin, depression.

## Abstract

Stress is the major predisposing and precipitating factor in the onset of depression which is the most significant mental health risk for women. Behavioral studies in animal models show that female sex though less affected by an acute stressor; exposure to repeated stressors induces coping deficits to impair adaptation in them. A decrease in the function of 5-hydroxytryptamine (5-HT; serotonin) in the hippocampus and an increased function of the 5-HT-1A receptor in the raphe nucleus coexist in depression. Pharmacological and neurochemical data are relevant that facilitation of serotonin neurotransmission via hippocampus due to desensitization of somatodendritic 5-HT1A receptors may lead to adaptation to stress. The present article reviews research on sex related differences of raphe-hippocampal serotonin neurotransmission to find a possible answer that may account for the sex differences of adaptation to stress reported in preclinical research and greater incidence of depression in women than men.

## 1. INTRODUCTION

It is well accepted that males and females can behave differently but how much of that difference is attributable to genetic, environmental and neurobiological factors has often been a matter of debate [1-3]. Evidence suggests that the effects of sex hormones on brain organization occur so early in life that from the start the environment is acting on differently wired brains in the two sexes. Human studies on sex differences of brain function are often supported by preclinical research suggesting that the differences are attributable to biological differences between males and females because there are few environmental or socio-cultural factors to consider in preclinical research.

Women are at least twice as likely as men to suffer from depression and anxiety [4-6]. These sex differences are seen in different countries and cultures, suggesting a biological basis. Evidence from animal studies also suggests that behavioral and neurobiological effects of stress are sexually dimorphic. However, despite great interest in this area [7], mechanisms that may contribute to this striking sex difference have remained elusive.

A dysfunctional 5-hydroxytryptamine (5-HT; serotonin)-ergic system is a vulnerability factor for major depressive disorder and other forms of affective illnesses [2]. At least 14 different types and subtypes of serotonin receptors have been identified [8]. A number of these receptors such as 5-HT-2 [9, 10], 5-HT-3 [11] and 5-HT-1A [12] receptors play a role in the genesis of psychiatric illnesses. Studies on animal models show that raphe hippocampal serotonin neurotransmission and its regulation *via* 5-HT-1A receptors can explain vulnerability or resistance to stress stimuli. The 5-HT-1A receptor which is a key mediator of serotonergic signaling in the central nervous system is also implicated in the mechanism of action of selective serotonin reuptake inhibitors (SSRIs) [13-16], while some studies indicate that sex may moderate the response to antidepressants with women exhibiting a preferential response to SSRIs compared to tricyclic antidepressants (TCAs) [17].

Cell bodies of serotonin containing neurons are located in the raphe nuclei in the brain stem. The 5-HT-1A receptor is a G-protein-coupled receptor widely distributed in regions that receive serotonergic input from the raphe nuclei: the frontal cortex, septum, amygdale, hippocampus and hypothalamus [18, 19]. It also serves as somatodendritic autoreceptor of raphe nuclei reducing the firing rate of serotonergic neurons [8, 20-22]. The hippocampus has been extensively studied with regard to stress, depression and antidepressant action [23-25]. Sex related differences of raphe-hippocampal serotonin neurotransmission with a particular focus on 5-HT-1A receptors are accumulated in the present review as this may account for the gender differences of adaptation to stress and greater incidence of depression in women than men.

## 2 STRESS, DEPRESSION AND THE GENDER DIFFERENCE

The hypothesis that stress is the major precipitating factor in the onset of depression is consistently supported by clinical and preclinical studies [26-28] showing the relationship between previous traumatic stressful event (predisposing factor) and subsequent other stressor (precipitating factor). Both physical and psychological stressors have been shown to lead to the onset of a depressive episode. Sex in genetically predisoposed subjects may result in depression [29]. Studies in female twins show a clear interaction between genetic loading and exposure to a recent stressful life event in the precipitation of depressive episode [30].

In a study of 4,856 individuals (53% female) experiencing depression, it has been seen that different types of adverse life events are associated with different depressive symptoms profile [31]. In a follow-up study of over 7 years in 266 middle aged women, without a history of major depression at base line, 15.8% women met criteria for major depression [32]. These researchers reported that lifetime history of anxiety disorder and very stressful life events are important contributing factors in the onset of first episode of major depression.

Major depressive disorder is two times more prevalent in women than in men [4, 6, 33, 34]. The mean age of onset, the overall course of depression and the risk for chronic or recurrent depression does not differ between sexes [35], although some studies suggest that women have a higher rate of recurrent depression and slower recovery from a depressive episode [36].

Women are approximately three times more likely to develop depression in response to stress because they experience more stressful events [30, 37-39]. Women report more depressive symptoms than men, with an emphasis on worthlessness, decreased sexual interest, guilt feelings, insomnia, anxiety, and gastrointestinal symptoms [36, 40]. This prominent gender difference in depression begins in adolescence, prior to which the incidence of major depression is equal in girls and boys, suggesting the potential role of female sex hormones in female depression vulnerability [41]. Many depressed women also exhibit anxiety symptoms, and it has been suggested that women may be more likely to suffer from mixed anxiety-depressive disorder [42, 43]. Polymorphic variations in 5-HT transporter, MAO-A or 5-HT receptor may be involved in the sex related differences of adaptation to stress [29].

## 3 STRESS CONTROLLABILITY AND LEARNED HELPLESSNESS IN ANIMAL MODELS

Based upon the clinical evidence that links stressful life events with depressive episodes several animal models exhibiting stressor controllability and learned helplessness have been developed [44-47]. The most common animal model of 'stress and coping' is that of 'learned helplessness' [48, 49] in which animals are exposed to either controllable or uncontrollable stressful events and later, they are tested on a new task in which all animals are given the opportunity to control the stressor, usually by escape. In most reports, animals that are exposed to uncontrollable stressful events do not learn to escape during testing on the new task [50, 51]. This behavior has been equated with a sense of 'giving up', experienced by humans with major depression [52].

Animal models of depression should fulfill three major criteria [47]. The first criterion “face validity” assesses how well the symptoms observed in animals resemble those in human patients. The second criterion “predictive validity” addresses the question how well animals in the model respond favorably to the same drugs as human do under the same treatment conditions. The third criterion “construct validity” assesses to what extent the model is consistent with the theoretical rationale.

The learned helplessness paradigm was not developed to provide an animal model of depression or anxiety but it was shown in later studies that the model is sensitive to both antidepressants [53, 54] and anxiolytics [55-58]. Implications for the learned helplessness paradigm as an animal model of either depression or anxiety have been discussed [45,59]. The paradigm is widely used to understand neural mechanism and degree of behavioral adaptation to an uncontrollable stressor [60-63].

Animals exposed to other unpredictable and uncontrollable stressor e.g. restraint stress, elevated platform and forced swimming also show coping deficits for aversive but escapable situations [64, 65]. Chronic mild stress also causes behavioral changes in animals that parallels symptoms of depression [66, 67].

## 4 SEX RELATED DIFFERENCES IN ADAPTATION TO STRESS

Although learned helplessness is an established model for clinical depression and anxiety, and has been investigated for about 40 years, only few studies have used female animals in the learned helplessness paradigm. The female preponderance of depression is also, heretofore, not been consistently reflected in animal models (Table **1**). Surprisingly, evidence from animal studies suggests that females are relatively resistant to the behavioral effects of an acute stress compared to males.

Exposure to inescapable foot shock disrupted shuttle box-escape performance of males, whereas, escape performance of females was unaffected [68]. Escape latencies increased in both males and females but the increases were greater in male rats [69]. In the elevated plus-maze also, exposure to inescapable foot shock resulted in suppression of “total number of arm entries” and “rearings” in males but not in females [70]. In addition “time on open arms” was reduced in both sexes, but this effect was stronger in males than in females. No sex based difference was found in learned helplessness behavior in another study [71] which reported that females to be more vulnerable than males to stress-induced elevations in homocysteine but not escape deficits.

Exposure to controllable stress (escapable foots hock) alleviated the expression of helplessness behavior in both females and males, but females learned to escape more rapidly than did males [72]. Moreover, modulation of controllability i.e exposure to inescapable foot shock produced helpless behavior in males but females were less likely to become helpless [72].

Male rats were more vulnerable to restraint stress than that of the female rats because open field behavior of female rats was less affected by a single 2h restraint stress than that of the male rats, though food intake was comparably decreased [73]. Male animals exhibited more immobility than females in the forced swimming test [3, 74-78] but young female rats were more vulnerable than males in an open space swim test [79].

Male rats were also more vulnerable to a mild lipopolysaccharide (LPS) challenge than that of female rats as assessed in both forced swim and open field test [80] because LPS challenge decreased open field activity in male but not female rats. LPS-treated female rats coped better with the stressful forced swimming procedure, as evidenced by an increase in swimming duration while in males swimming duration was not altered by LPS administration [80].

In striking contrast female but not male rats exhibited deficits of open field behavior after exposure to repeated restraint stresses [64, 73, 81, 82]. Female rats were more vulnerable to chronic mild stress as depicted by the disruption of sucrose intake and decreases of open field activity [66]. But in response to an additional forced swim test, females previously exposed to chronic mild stress, were found to cope better than males. The sex differences in helplessness behavior were not dependent on the presence of sex hormones in adulthood, because neither ovariectomy of females nor castration of males abolished those [83].

**Fig. (1):**
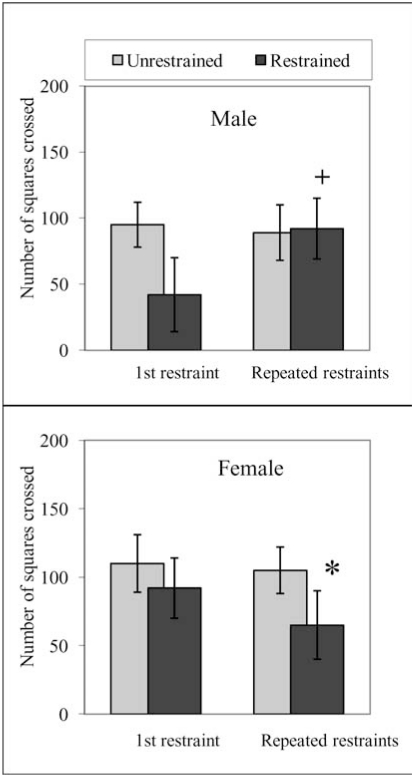
Activity of male and female rats in an open field 24 h after a single (2h) or repeated (2h/day for 5 days) restraints. Male (body weights 200-250 gm) and female (body weights 190-230) animals were restrained for 2h on wire grids and activity in an open field was monitored 24 h after the termination of the 1^st^ or 5^th^ restraint period as described by Haleem & Parveen (1994). Values are means ± S. D. (n=6). Significant differences by Newman Keuls test: *P<0.01 from respective unrestrained animals; +P<0.01 from 1^st^ day (2h/day) restrained animals, following two way ANOVA. (Haleem unpublished data).

In general sex related studies on animal models show that female performance though better than males if exposed to an acute stressor but repeated or chronic exposure induces coping deficits to impair adaptation making female sex more vulnerable to depression. Data in Fig. (**1**) (Haleem unpublished data), similar to previously reported studies [64, 73, 81] show that male and female rats exposed to 2h restraint stress exhibited a decrease in open field activity monitored on the following day but the decreases were smaller and not significant in female rats. These differences were not attributable to the effects of entrained oestrus cycles in females as these were randomly distributed [73, 81]. Conversely, following repeated (2h/day for 5 days) exposure to restraint stress the deficits of open field exploration were present in female but not male rats (Fig. **1**).

## 5 RAPHE-HIPPOCAMPAL SEROTONIN NEUROTRANSMISSION AND ADAPTATION TO STRESS

Early indications that central serotonin system may be involved in responses to stress came from studies on male rats demonstrating that acute stress procedures like immobilization, forced swimming and cold exposure increased brain 5-HT metabolism [84, 85]. Since then evidence has accumulated that 5-HT turnover is enhanced by following exposure to various stressors including exercise and foot shock although brain levels of 5-HT are not always altered [1, 4, 86-88]. It has been also shown that stress-induced increases of brain serotonin are caused by an increase in the availability of tryptophan [86, 88], the precursor of 5-HT, or an increase in the activity of tryptophan hydroxylase [89-92], the rate limiting enzyme of 5-HT biosynthesis. Microdialysis studies showed an increase in extracellular levels of serotonin [93-95] in different areas of the brain following exposure to different types of stressors.

A role of hippocampus in responses to stress, first reported from our laboratory [90], also emerged from studies on male rats. We found that acute exposure to an episode of 2h restraint stress increased 5-HT turnover in the hypothalamus, midbrain and cortex but the increases did not occur in the hippocampus [90]. Conversely, repeated daily exposure to 2h/day restraint, which produced behavioral adaptation, increased 5-HT turnover in the hippocampus only and not in other brain regions. It was suggested that an increase in serotonin neurotransmission *via* hippocampus is involved in adaptation to stress.

Later studies, performed on male animals, also consistently showed that hippocampus may mediate adaptation to severe inescapable stressor by the facilitation of serotonergic neurotransmission (Table **2**). Acute exposure to an elevated platform enhanced 5-HT overflow in the prefrontal cortex but not dorsal hippocampus whereas repeated daily exposure to the same stressor increased extracellular 5-HT in the dorsal hippocampus but not the prefrontal cortex [65]. In another study rats received inescapable foot shock and were tested in a shuttle box 24 h later. Pre stressed animals exhibited impairment of escape responses. This effect was prevented by bilateral intra hippocampal injection of zimelidine, a serotonin reuptake blocker but not by desipramine, a noradrenaline reuptake blocker [96]. Neurogenesis in the dentate gyrus of the hippocampus was enhanced by the activation of serotonin receptors [97]. It was suppressed by stress and the suppression prevented by 5-HT-1A receptor agonists [98].

The synthesis and release of 5-HT in all brain regions including the hippocampus [20-22, 99] is under the control of an effective feedback mechanism involving the stimulation of 5-HT-1A receptors located on the soma and dendrites [100] of the serotonergic neurons in the raphe nucleus. 5-HT1B receptors located at the terminal ends of the serotonergic neurons [101] also control the release of 5-HT *via* a feedback mechanism [102]. Studies on the mechanism of action of selective serotonin reuptake inhibitors (SSRIs) and other antidepressants showed that repeated administration of these drugs increased 5-HT neurotransmission by either decreasing the sensitivity of presynaptic receptor or increasing the sensitivity of postsynaptic 5-HT-1A receptor in the dorsal hippocampus [13, 14, 16, 103]. Consequently it was suggested that a decrease in the function of the 5-HT in the hippocampus and an increased function of the 5-HT-1A receptor in the raphe nucleus coexist in depression.

**Fig. (2):**
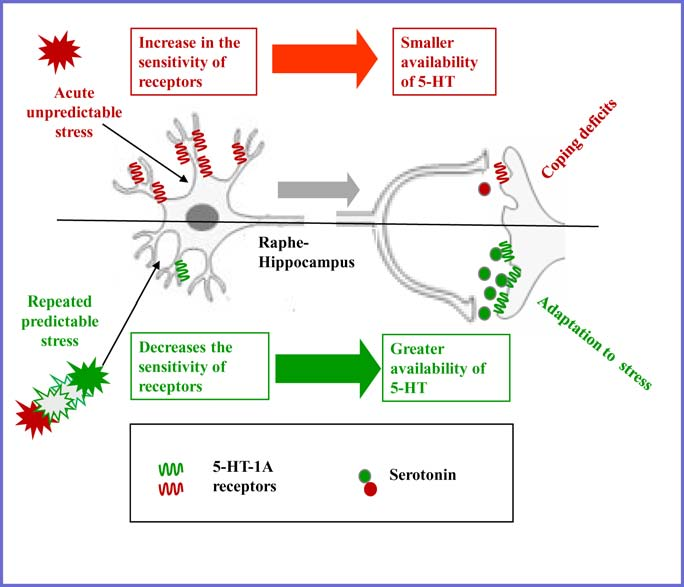
Attenuation (red) and enhancement (green) of raphe-hippocampal serotonin neurotransmission regulated by somatodendritic 5-HT-1A receptors.

Studies using learned helplessness model of anxiety/depression and chronic stress model of depression also support the role of 5-HT-1A receptor in adaptation to stress. Thus, rats adapted to repeated restrain stress schedule of 2h/day for 5 days exhibited a decrease in the sensitivity of somatodendritic 5-HT-1A [60, 104] and terminal 5-HT-1B [105] receptors: an effect similar to antidepressant like effect. It was suggested that a decrease in the negative feedback control due to desensitization of auto receptors increases the availability of 5-HT in terminal regions to help cope the stress demand and produce adaptation to stress (Fig **2**). Conversely, exposure to inescapable but not escapable stressors sensitized serotonergic neurons in the raphe region to subsequent input [85]. Acute exposure to 2h restraint stress [106] as well as long term starvation [107] increased the responsiveness of somatodendritic 5-HT-1A receptor to decrease serotonin neurotransmission particularly *via* raphe-hippcampal pathway (Fig. **2**). A decrease in the density of 5-HT-1A receptor in the hippocampus also occurred in rats exposed to restraint stress [98, 108, 109]. Rats exposed to different mild to moderate stressors every day, therefore making the daily stress exposure unpredictable exhibited a significant decrease in 5-HT-1A mRNA and 5-HT-1A receptor binding in the hippocampus [23].

It may be argued that a desensitization and super sensitization respectively of autoreceptors would be expected to increase and decrease the availability of 5-HT in all brain regions innervated by serotonergic neurons and not particularly in the hippocampus [90]. An explanation to this could be that postsynaptic 5-HT-1A receptors also control the synthesis and release of 5-HT *via* feedback mechanism. Hippocampus is enriched with 5-HT-1A receptor [110] and receives serotonergic innervations from median raphe [111]. Many innervated areas project back to raphe nuclei and these are interconnected [112]. It is therefore possible that postsynaptic 5-HT-1A receptors also alter the median raphe nucleus 5-HT neuronal firing [15]. It is also possible that the effects are mediated *via* stress-induced release of corticosteroid hormones. Hippocampus is enriched with high affinity mineralocorticoid receptors and lower affinity glucocorticoid receptors at which corticosteroids bind to alter 5-HT-1A receptor mediated responses, reviewed by Joel, [113].

## 6 SEX-DIFFERENCES IN RAPHE-HIPPOCAMPAL SEROTONIN NEUROTRANSMISSION

If facilitation of serotonin neurotransmission due to desensitization of somatodendritic 5-HT-1A receptor increasing the availability of 5-HT in the hippocampus mediates adaptation to stress, the sex differences of somatodendritic 5-HT-1A receptors become important. Sexual dimorphism in the serotonin was first reported in early 1960’s [114]. An increasing amount of later work supported the view that central serotonin metabolism synthesis and functional responses are greater in female than male rats [20, 115-117]. It was also observed that sex differences of 5-HT were particularly larger in the hippocampus [20].

Sex differences also occur in the regulation of serotonin neurotransmission *via* 5-HT-1A receptors [118]. Expression of serotonin-1A receptor messenger RNA was greater in males in the hypothalamus and amygdala, and less in males in the hippocampus [119]. The concentrations of 5-HT and 5-HIAA were greater in the hippocampus of female than male rats. The 5-HT-1A agonist 8-hydroxy-2 (di-n-propylamino) tetralin caused comparable decreases of 5-HT and 5-HIAA in both sexes in the hypothalamus, cortex and midbrain except the hippocampus where the decreases were twice as large in the females as in males [20]. It suggests that the sensitivity of 5-HT-1A receptors that control the availability of 5-HT in the hippocampus (Fig. **2**) is greater in female sex.

A few studies have examined sex influence on the role of hippocampus in responses to stress. Female in proestrus exhibited greater density of dendritic spines in the area CA1 of the hippocampus than males [120]. In response to acute stressful event of intermittent shocks, spine density was enhanced in the male hippocampus but reduced in the female hippocampus. Effects of early experience on the dendritic structure of dentate gyrus were also sexually dimorphic [121]. Thus female rats raised in an enriched environment displayed increased dendritic bushiness relative to males raised in the same environment. Conversely, neonatal handling resulted in an increase in postsynaptic serotonin neurotransmission in the hippocampus of male rats but decreased it in females [122]. LPS treatment induced a female-specific enhancement of 5-HIAA levels in the hippocampus and some other regions [80].

In a study of sex influence and isolation housing on 5-HT-1A receptor binding female mice displayed lower postsynaptic 5-HT-1A receptor binding compared to males in the hippocampus. Subsequently, following 6 weeks isolation housing 5-HT-1A receptor binding was further increased in males but not in females [123]. Conversely, forced swimming was found to decrease 5-HT-1A receptor binding in the hippocampus of female but not male rats [124]. Serotonin-1A mRNA, protein and binding sites, were greater in the hippocampi of pre-pubertal female than male rats. These were decreased more by neonatal handling and the decreases were greater in female sex [122].

Sex related studies therefore show that male and female animals have different levels of serotonin neurotransmission *via* raphe- hippocampal pathway under unstressed condition, which can respond in opposite directions to the same stressor. Females have greater serotonin content but an exaggerated feedback control over raphe-hippocampal serotonin neurotransmission *via* 5-HT-1A receptors making this sex more vulnerable to depression.

It is worth considering that serotonin functions are modulated by corticosteroids [113, 125, 126] while stress-induced [81] as well as 5-HT-1A agonist-induced [82] increase of plasma corticosterone, the principal corticosteroid secreted by the rat adrenal gland, are greater in female than male rats suggesting an important role of circulating corticosteroids in the sex related differences of raphe hippocampal serotonin neurotransmission in adaptation to stress. Influence of MAO-A genotype on 5-HT-1A receptor availability or polymorphic variations of 5-HT transporters [108] may well be involved in these sex differences of 5-HT-1A expression.

5-HT-1A receptor dependent responses are also modulated by estrogen. Thus, acute estrogen treatment prevented 5-HT1A receptor-induced disruption of Prepulse inhibition in healthy women [127]. Although, estrogen treatment to ovariectomized rats had no effect on the number or affinity of 5-HT_1A_ binding sites labeled with [^3^H]8-OH-DPAT but 5-HT_1A_-mediated inhibition of adenylate cyclase selectively increased in the hippocampus [128]. A role of glutamate receptors in the sex related differences of adaptation to stress is also possible because antidepressant like activity of chromium chloride in the forced swim test in mice was inhibited by antagonists of glutamate receptors as well as antagonists of 5-HT-1A receptors [129].

## 7 POSSIBLE CLINICAL RELEVANCE

There is wealth of clinical evidence supporting sex difference in 5-HT-1A receptor function. Investigations have also been made to show that variation in 5-HT-1A expression is genetically mediated [130, 131].

Age related sexual dimorphism of 5-HT-1A receptor binding potential was initially observed in various brain tissues obtained from autopsy subjects. In this study men exhibited a significant age dependent decrease in the dissociation constant (Kd) for 5-HT-1A receptor binding in the occipital cortex; in women maximum binding capacity (Bmax) decreased with aging in the parietal cortex and hippocampus [132].

Parsey *et al.* [133] did not find an age related decrease in 5-HT-1A binding potential in healthy men or women. However, they found higher 5-HT-1A binding potential in the dorsal raphe and many forebrain regions of women than men. Conversely, 5-HT-2 receptor binding capacity was higher in healthy men than healthy women [134]. Staley *et al.* [135] observed higher 5-HT transporter availability in healthy women than men, but lower 5-HT transporter availability in depressed women than depressed men [136]. Javanovic *et al.* [137] also observed that compared to healthy men healthy women had significantly higher 5-HT-1A receptor but lower 5-HT transporter binding potential in a wide array of cortical and subcortical brain regions but in the follicular phase, women did not differ from men in the 5-HT1A receptor binding [138].

Several strands of evidence have emerged that specifically implicate 5-HT-1A receptors in depression and therapeutic effects of antidepressant drugs. Men and women patients with major depression exhibited attenuation of 5-HT-1A receptor mediated neuroendocrine and hypothermic responses reflecting a decrease in the effectiveness of postsynaptic and somatodendritic 5-HT-1A receptors respectively [46, 139, 140]. A decrease in 5-HT-1A binding potential has been also observed in the multiple brain areas including raphe region of men and women patients with major depression and bipolar disorder [141-143]. In another study, patients with major depression who have never been exposed to medication were found to have higher 5-HT-1A receptor binding compared to the depressed patients with a history of medication and control [133] suggesting the 5-HT-1A binding potential to be affected by medication. Higher 5-HT1A binding potential in the raphe and hippocampus in bipolar depressed males but not in bipolar depressed females has been also reported [68].

Currently the most common class of effective antidepressants is SSRIs that acts by selectively blocking the high affinity reuptake of serotonin. Approximately 78% of the prescribed SSRIs are given to women [5], while some studies indicate that sex may moderate the response to antidepressants with women exhibiting a preferential response to SSRIs compared to TCAs. Investigations addressing gender differences in response to SSRI (sertraline) and imipramine treatment in male and female patients with chronic depression have reported that premenopausal women had a favorable response to sertraline than to imipramine [17, 144]. Postmenopausal women exhibited similar response to the two medications and men exhibited a more favorable response to impramine than to sertraline. Martenyi *et al.* [145] compared treatment efficacy of SSRI (fluoxetine) and SNRI (maprotiline) in men and women patients of unipolar depression. They found a significant difference between treatment groups in females but not in males. Amongst females the difference was significant in women aged <44 years but not >44 years suggesting that women in their reproductive period are more responsive to SSRIs than SNRIs. In a recent study, Young *et al.* [146] have reported that women have a better response to the SSRI citalopram than men, which may be due to sex-specific biological differences particularly in serotonergic systems.

## CONCLUSION

The evidence accumulated in the present article suggests that raphe hippocampal serotonin neurotransmission and its regulation by 5-HT-1A receptors has an important role in the sex related differences of adaptation to stress. Greater 5-HT neurotransmission *via* postsynaptic 5-HT-1A receptors in the hippocampus makes female sex more resistant to an acute stressor. On the other hand, greater efficacy of feedback control over 5-HT synthesis and release mediated *via* 5-HT-1A receptors could impair adaptation making the female sex more vulnerable to repeated and/or chronic stressors. In the quest to understand the mechanism of sex related differences in adaptation to stress, the role of raphe-hippocampal serotonin neurotransmission and its regulation by 5-HT-1A receptors can only be a small part of a big picture. The mechanisms through which estrogen and glucocorticoids can modulate serotonin neurotransmission and functional polymorphisms in the 5-HT transporter gene are also worth considering for an understanding of sex related differences of adaptation to stress. Despite heightened complexity it implies that the issue of sex related differences of brain function is not less important because it may provide ways to understand novel mechanisms of brain function.

## Figures and Tables

**Table 1:** Sex Related Differences in Stress-Induced Behavioral Deficits Stressor

Stressor	Behavior	Behavioral Deficits	References
Forced swimming	Latency and duration of immobility	Male > Female	[75-79]
Open space swimming	Latency and duration of immobility	Young Female > Young Male	[80]
Escapable shock Inescapable shock	Escape latency Escape impairment	Male> Female Male > Female Male = Female	[73] [69-71, 73] [72]
Chronic mild stress	Disruption of sucrose intake & open field activity	Female > Male	[67, 84]
Chronic mild stress + Forced swimming	Latency and duration of immobility	Male > Female	[67, 84]
Single 2h restraint	Open field activity	Male > Female	[74, 82]
Repeated restraint	Open field activity	Female > Male	[74, 82]
Lipopolysaccharide challenge	Open field activity & Forced swim test	Male > Female Male > Female	[81] [81]

Stressors to which female sex is more vulnerable are highlighted.

**Table 2:** Evidence that Facilitation of Serotonin Neurotransmission in the Hippocampus is Involved in Adaptation to Stress

Challenge	Response	Hippocampal 5-HT	References
Restraint stress	Decrease in food intake & open field activity	5-HT increased in many brain regions except the hippocampus	[91]
Repeated restraint	Normal food intake & open field activity	5-HT increased only in the hippocampus	[91]
Acute exposure to elevated platform	Increase in plasma corticosterone	Extra cellular 5-HT increased in the frontal cortex but not the hippocampus	[66]
Repeated daily (10 days) exposure to elevated platform	Normal plasma corticosterone response	Extra cellular 5-HT increased in the hippocampus but not the frontal cortex	[66]
Inescapable foot shock	Escape impairment in shuttle box	The behavioral deficit normalized with bilateral intra hippocampal serotonin reuptake inhibitor	[97]
Subordination stress	Neurogenesis	Stress-induced suppression of neurogenesis in the hippocampus prevented by 5-HT-1A agonists	[98-99]
Forced swimming	Immobility	Decreased 5-HT-1A receptor binding in the hippocampus	[125]
Restraint stress	Feedback control over 5-HT	Exaggerated feedback control over hippocampal 5-HT	[107]
Restraint stress	Density of 5-HT-1A receptor	5-HT-1A receptor binding decreased in the hippocampus	[99, 110, 119]
Unpredictable, mild to moderate stressors	5-HT-1A mRNA	5-HT-1A expression decreased in the hippocampus	[23]
Long term administration of SSRIs	Feedback control over 5-HT	Smaller feedback effects over hippocampal 5-HT	[13, 14, 104]
